# The Mechanism of Selective Recognition of Lipid Substrate by hDHHC20 Enzyme

**DOI:** 10.3390/ijms232314791

**Published:** 2022-11-26

**Authors:** Irina S. Panina, Nikolay A. Krylov, Anton O. Chugunov, Roman G. Efremov, Larisa V. Kordyukova

**Affiliations:** 1 Shemyakin-Ovchinnikov Institute of Bioorganic Chemistry, Russian Academy of Sciences, 117997 Moscow, Russia; 2 International Laboratory for Supercomputer Atomistic Modelling and Multi-Scale Analysis, National Research University Higher School of Economics, 101000 Moscow, Russia; 3 Moscow Institute of Physics and Technology, State University, Dolgoprudny, 141701 Moscow, Russia; 4 Belozersky Institute of Physico-Chemical Biology, Lomonosov Moscow State University, 119991 Moscow, Russia

**Keywords:** DHHC-acyltransferase, hDHHC20, S-acylation, S-palmitoylation, lipid selectivity, molecular dynamics, computer modeling, intermolecular interactions

## Abstract

S-acylation is a post-translational linkage of long chain fatty acids to cysteines, playing a key role in normal physiology and disease. In human cells, the reaction is catalyzed by a family of 23 membrane DHHC-acyltransferases (carrying an Asp-His-His-Cys catalytic motif) in two stages: (1) acyl-CoA-mediated autoacylation of the enzyme; and (2) further transfer of the acyl chain to a protein substrate. Despite the availability of a 3D-structure of human acyltransferase (hDHHC20), the molecular aspects of lipid selectivity of DHHC-acyltransferases remain unclear. In this paper, using molecular dynamics (MD) simulations, we studied membrane-bound hDHHC20 right before the acylation by C12-, C14-, C16-, C18-, and C20-CoA substrates. We found that: (1) regardless of the chain length, its terminal methyl group always reaches the “ceiling” of the enzyme’s cavity; (2) only for C16, an optimal “reactivity” (assessed by a simple geometric criterion) permits the autoacylation; (3) in MD, some key interactions between an acyl-CoA and a protein differ from those in the reference crystal structure of the C16-CoA-hDHHS20 mutant complex (probably, because this structure corresponds to a non-native dimer). These features of specific recognition of full-size acyl-CoA substrates support our previous hypothesis of “geometric and physicochemical selectivity” derived for simplified acyl-CoA analogues.

## 1. Introduction

S-palmitoylation (S-acylation) is a versatile post-translational modification of peripheral and integral membrane proteins by palmitate (C16:0) or other long-chain fatty acids, which are attached to cysteine residues via thioester bond. Unlike other well-known lipid modifications of cellular proteins (such as N-myristoylation, N-/O-acylation [1]), S-palmitoylation is usually a reversible process [1,2], which allows the dynamic regulation of the local hydrophobicity of acylated proteins. The attached fatty acid residue not only increases the affinity of soluble proteins to the membrane, but also affects the conformational rearrangements of integral membrane proteins and changes the intra- and intermolecular protein–protein interactions [3]. Among thousands of S-palmitoylated proteins many are responsive for signal transmission, reception, ion conduction and transcription regulation, which is often coupled to cancer progression or neurodegenerative diseases [4,5]. Unlike cellular proteins, S-acylation of proteins of pathogenic enveloped viruses is irreversible [6]. It may affect the topography of the protein within the membrane [7], is involved in the membrane fusion reaction and/or assembly of progeny virions [8,9], and, therefore, is required for the reproduction of functionally competent viruses [10].

Palmitoylation is catalyzed by a family of integral membrane enzymes—DHHC-acyltransferases, which contain the characteristic catalytic motif Asp-His-His-Cys (DHHC). The catalytic process consists of two stages: (1) autoacylation using an intracellular fatty acid donor—acyl-CoA; (2) transfer of the acyl chain from the acyl enzyme to the protein substrate [11]. A family of human DHHC acyltransferases (hDHHC) comprise 23 enzymes that differ in their specificity to the protein substrate, as well as in the length of the transported acyl chain. Despite the important role of palmitoylation in the functioning of many cellular and viral proteins, the molecular aspects of DHHC acyltransferases’ substrate selectivity remain unclear.

hDHHC20 is one of the most functionally characterized proteins of the family and the only human DHHC enzyme with a known spatial structure [11]. hDHHC20 substrates are, for example: epidermal growth factor receptor (EGFR) [12] and fusion proteins of important pathogenic viruses, such as influenza virus hemagglutinin [13] and spike (S) protein of SARS-CoV-2 [14,15,16]. The 3D-structure of hDHHC20 includes four transmembrane ɑ-helices (TMH), which form a teepee-like structure with the bottleneck at the extracellular part of the membrane; and a large intracellular (cytoplasmic) domain [11]. The catalytic DHHC motif is located at the membrane-cytosol interface, at the very bottom of the cavity where the fatty acid residue is accommodated during the autoacylation step. Biochemical studies revealed that hDHHC20 predominantly transfers C16:0 chains, and this selectivity may be shifted toward shorter myristate (C14:0) or longer stearate (C18:0) residues by an artificial introduction of S29F or Y181A mutations, respectively [11].

At the moment, the mechanism of hDHHC20 selectivity to the lipid substrate at the atomic level is largely unknown [17]. In our previous work [18], using computer modeling, we studied three sub-stages of hDHHC20 autoacylation: (1) an isolated enzyme without a substrate in the membrane; (2) pre-acylation—the moment prior to the chemical reaction, when the enzyme and substrate are located in close proximity to each other; and (3) post-acylation—right after the chemical reaction, when the fatty acid residue is transferred from acyl-CoA to the catalytic Cys^156^ residue of the enzyme. A shortened acyl-CoA analogue—acyl-β-mercapto-ethylamine (acyl-MEA) with a less voluminous headgroup—was used in that study, as the complex with the full-size acyl-CoA [19] was unavailable at that moment and was released shortly after our publication [18]. It was found that the inner cavity of the protein is formed by the uptake of the lipid substrate or the membrane phospholipid’s acyl chain. In the absence of a lipid tail inside the cavity, DHHC protein exhibits decreased density, but not a distinct void space. It has also been hypothesized that hDHHC20 lipid selectivity is determined by the geometric and physico-chemical characteristics of the enzyme cavity: just above the center of the membrane, the TMHs of the protein intersect, preventing the substrate’s lipid tail from penetrating deeper into the cavity. The terminal methyl groups of all acyl chains, regardless of their length, line up against the cavity’s “ceiling”. As a result, the sulfur atom in the catalytic residue Cys^156^ and the reactive carbon atom in the acyl-CoA should be located at different distances. The optimal distance for the chemical reaction to proceed is assumed for C16-CoA.

In this paper, using the molecular dynamics (MD) simulations, a pre-acylation system is studied in more detail, including not a shortened [18], but a full-size acyl-CoA (based on the most recent crystal structure [19]), bringing the model system closer to the native one. The obtained data confirm the earlier hypothesis that hDHHC20 lipid selectivity depends on the geometric and physicochemical characteristics of the enzyme’s cavity, explains the maximal fitness and “reactivity” of the C16 substrate for hDHHC20 and lightens the way for in silico identification of the best substrate for the uncharacterized DHHC enzymes.

## 2. Results

We studied hDHHC20 in a complex with full-sized acyl-CoA molecules of lengths C12, C14, C16, C18 and C20, each in three replicas (see Table 1 for systems description). In these MD calculations, the enzyme structure remained relatively stable: averaged over all trajectories the root-mean–square deviation (RMSD) of Cα-atoms with respect to the start was 2.4 ± 0.6 Å. At the same time, in a few runs with the acyl-CoA of suboptimal length (less than C16—e.g., C12 and C14), acyl chains left the protein cavity and immersed into the lipid bilayer, keeping the CoA head bound to the cytosolic side of the enzyme. Additionally, in one MD run with C14-CoA, a subsequent sequence of “exit–entry–exit” events was observed. It is important to note that earlier we observed a similar dissociation of the C14-MEA complex [18], which may indicate, on the one hand, a lower protein affinity for suboptimal tails length; and, on the other hand, a dynamic and reversible nature of acyl substrate binding by the DHHC enzymes. Meanwhile, among three replicas there was always at least one trajectory with an acyl chain securely embedded in the enzyme’s cavity, which was assumed as “representative” (and subscribed with the ^(1)^ index in Table 1) and used for all the results below, if other is not specified.

**Figure 1:**
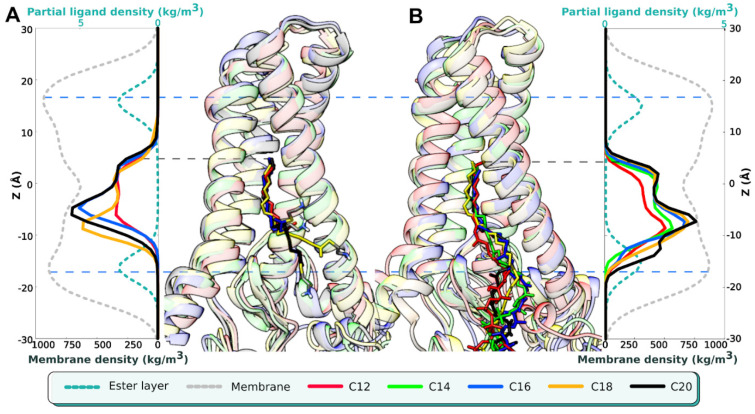
Positioning of the unbound hDHHC20 ligands along the membrane normal (axis Z): acyl-MEA (**A**) and acyl-CoA (**B**). Middle part: aligned conformations from the final MD-snapshots of hDHHC20 with acyl substrates of different length (colored according to the legend). Left and right panels: MD-derived partial density profiles for the respective acyl chain (data for acyl-MEA are taken from [18]). Z = 0 corresponds to the bilayer center. Protein is shown in a ribbon presentation. Lower axis describes the partial POPC density, upper—MEA/CoA acyls. Calculations for (**B**) performed on a set of representative trajectories (Table 1).

One of the major findings of our previous work [18] was that regardless the chain length, the terminal methyl group of the unbound acyl-MEA inside the enzyme hits the “ceiling” of the cavity—in particular, shorter C12- and C14-chains slipped entirely into the cavity. Here, we reproduce this result on full-size acyl-CoA molecules: regardless of the initial position, their terminal methyl groups penetrate at the same depth. Figure 1 compares these findings for mimetics (acyl-MEAs) from the earlier study [18] and for acyl-CoAs (this work): terminal methyl groups reach the same level of Z (coordinate along the membrane normal) ≈ 6 Å, in spite of different position of their heads. Unlike CoAs, small ethylamine heads of MEAs can be located both at the membrane-water interface and in the hydrophobic membrane layer, followed by twisting of acyl chains (Figure 1A); while a more massive and polar CoA head does not exhibit such a behavior, maintaining the acyl tails straight (Figure 1B). In such a way, terminal positioning of the methyl groups does not depend on the CoA head’s binding and always reaches the cavity ceiling. This may indicate that the low-density packing cavity inside the DHHC protein “sucks up” the adjacent lipid acyl chain, filling the empty space.

**Table 1:** Systems used in MD simulations *.

Name of MD Run	System Composition	<D_1_>, Å	D_1_>, Å	<α_BD_>, °
C12 ^(1)^ C12 ^(2)^ C12 ^(3)^	hDHHC20_1_/C12-CoA_1_/POPC_238_/H_2_O_24944_/Na^+^_64_/Cl^−^_69_	−2.7 ± 0.6 −11.4 ± 3.2 −11.5 ± 1.6	5 ± 0.5 5.7 ± 0.3 5.2 ± 0.7	40 ± 20 101 ± 23 52 ± 24
C14 ^(1)^ C14 ^(2)^ C14 ^(3)^	hDHHC20_1_/C14-CoA_1_/POPC_238_/H_2_O_24940_/Na^+^_66_/Cl^−^_71_	−2 ± 0.6 −9.4 ± 2.8 −5.3 ± 2.3	4.8 ± 0.5 4.4 ± 0.5 4.9 ± 0.6	42 ± 27 58 ± 30 75 ±42
C16 ^(1)^ C16 ^(2)^ C16 ^(3)^	hDHHC20_1_/C16-CoA_1_/POPC_238_/H_2_O_24935_/Na^+^_66_/Cl^−^_71_	−2.1 ± 0.3 −1.7 ± 0.4 −1.8 ± 0.6	5.1 ± 0.6 5.6 ± 0.3 4.7 ± 0.5	112 ± 34 35 ± 23 110 ± 54
C18 ^(1)^ C18 ^(2)^ C18 ^(3)^	hDHHC20_1_/C18-CoA_1_/POPC_237_/H_2_O_24939_/Na^+^_66_/Cl^−^_71_	−2.1 ± 0.8 −2.4 ± 0.9 −2 ± 0.7	5.3 ± 0.6 5.5 ± 0.5 5.4 ± 0.4	107 ± 41 71 ± 38 101 ± 32
C20 ^(1)^ C20 ^(2)^ C20 ^(3)^	hDHHC20_1_/C20-CoA_1_/POPC_237_/H_2_O_24940_/Na^+^_66_/Cl^−^_71_	−1.9 ± 0.3 −1.6 ± 0.6 −2.3 ± 0.9	5.4 ± 0.5 5.5 ± 0.5 5.1 ± 0.6	54 ± 40 61 ± 25 66 ± 38

**Figure 2:**
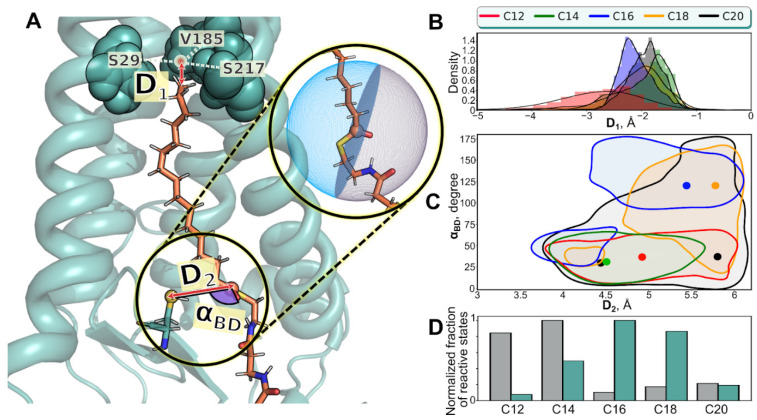
Positioning of the fatty acid residue inside the protein cavity and the complex reactivity during MD. (**A**). MD snapshot of hDHHC20 in complex with C16-CoA. Crucial residues of the protein ceiling (Ser^29^, Val^185^ and Ser^217^) are marked by their center of mass (*red circle*). Parameters D_1_, D_2_ and α _BD_ are illustrated. D_1_ is the ΔZ value for the tail terminal C-atom and the ceiling. D_2_ is the distance between the sulfur atom of the catalytic Cys^156^ residue (*yellow sphere*) and the carbonyl C atom of the acyl residue (*orange sphere*). α_BD_ is the Bürgi–Dunitz angle of the nucleophilic attack [20]; *semi-transparent blue hemisphere* in the *inset* illustrates the “right” area for Cys^156^’s sulfur atom to be treated as reactive. (**B**). D_1_ distribution for the representative set of trajectories (colored according to the legend). Time-dependencies of D_1_ and D_2_ are provided in Figure S3. (**C**). Contours of the high-density states in the coordinates (D_2_, α_BD_) for all MD trajectories from Table 1 (replicas joined). *Colored dots* show the positions of the maximas of these 2D-distributions. Full version of this analysis is provided in Figure S2. (**D**). Normalized fraction of the reactive states (in silico selectivity profile) for acyl-CoA of various chain lengths (*cyan bars*) compared to analogous profile, calculated with the “improper” α _BD_ (<90°) (*gray bars*).

* For each acyl tail length, three replicas were calculated. The representative one in each case is subscribed ^(1)^ and was used for most calculations, if the other is not stated. MD trajectory length is 500 ns. Additionally, MD-averaged (mean ± s.d.) values of D_1_, D_2_ and α _BD_ (see Figure 2 for parameters description) are provided (the two latter—averaged over reactive states).

The cavity ceiling and its interaction with tips of the ligand chain is clearly seen from the time-averaged MD contacts map (Figure S1): terminal C-atom of all substrates interacts preferentially with Ser^29^, Val^185^ and Ser^217^ triad (Figure 2A and Figure S1); sometimes Leu^213^ or Val^216^ are also involved. Trp^158^, which presumably forms the “entrance gate” to the enzyme cavity [18], interacts frequently with the lower part of the acyl tail. Interestingly, residues of TMH-2 interact with the substrate the least, despite the abundance of large aromatic residues facing the cavity.

To assess the fitness of the acyl tail for the enzyme’s cavity, we introduce parameter D_1_ (ΔZ between the acyl tail terminal C-atom and the center of mass of the ceiling; Figure 2A), which remains negative (Figure 2B), suggesting that the acyl tail always reaches the ceiling, but does not overcome it. D_1_ = −4.07 Å for the crystal structure.

Additionally to this “fitness”, we tried to describe the “reactivity”—readiness of the acyl-CoA molecule to transfer its acyl chain to the S atom of the catalytic C^156^ residue. MD simulations do not offer chemical reactivity, so we established a simplified criterion based on two parameters: D_2_ (distance between S atom of the catalytic Cys^156^ and carbonyl C atom of the acyl residue) and the Bürgi–Dunitz angle (α _BD_) of the nucleophilic attack [20] that has to occur for the transacylation to happen (see Figure 2A for these parameters description, Figure S3 for dynamic D_1_ and D_2_ values and Table 1 for MD-averaged D_1_ and D_2_ values, as well as α_BD_ values, averaged over the trajectories parts where D_2_ < 6 Å). There’s also second angle—Flippin–Lodge (α_FL_), which describes the coordination of chemical reaction more completely,—but here we limited ourselves with the mentioned couple of parameters.

For autoacylation to occur, a closer proximity between the reaction centers is required (e.g., D_2_ < 6 Å, characteristic for a Van der Waals contact), and α_BD_ has to be ≈107° for simple organic compounds, but may decrease to 89 ± 7° in enzymes [21]—anyway remaining obtuse. For our assessment, we treated as reactive conformations with D_2_ < 6 Å and α_BD_ > 90°. In the crystal structure, D_2_ = 6.6 Å, and α_BD_ = 127°, highlighting imperfect reactivity of this non-reactive mutant (hydroxyl O atom of mutant’s Ser^156^ was used for calculations). Figure 2C shows contours of the high-density states in the coordinates (D_2_, α_BD_), permitting identification of C16 system as reactive (most of the states have α_BD_ > 90°), C18 as partially reactive (just a portion of the states have α_BD_ > 90°) and C12, C14 and C20—as non-reactive (α_BD_ < 90°). In Figure 2D we show the calculated fraction of the reactive states (normalized to 100%) for all the trajectories, indicating that C16-CoA has the best potential (cyan bars), corroborating the experimental selectivity profile [11]. For the reference, we performed this calculation also for non-optimal α_BD_ < 90° (gray bars), where C16-CoA is given the least preference. This comparison exhibits that C16-CoA not only fits perfectly the hDHHC20 cavity, but also has maximal reactivity (as assessed by simple geometric criterion).

**Figure 3:**
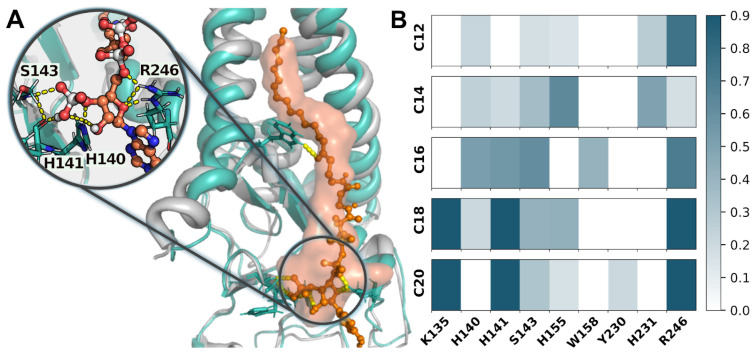
Key polar contacts of the acyl-CoA head with hDHHC20 during MD. (**A**). Comparison of the hDHHC20/C16-CoA complex from MD (hDHHC20—*green*; C16-CoA—*orange balls* & *sticks*) with the starting crystal structure (hDHHS20—*gray*; C16-CoA—s*emi-transparent orange surface*). Amino acid residues that bind acyl-CoA by h-bonds (*yellow dashes*) in MD structure, are shown with *sticks*. *Inset* shows the zoomed-in picture. (**B**). H-bonding map between hDHHC20 and acyl-CoA (representative trajectories are shown according to Table 1). Amino acid residues that form h-bonds with >10% lifetime in at least one trajectory are shown and colored according to the scale.

Acyl-CoA is an amphiphilic molecule. In complex with hDHHC20, its polar head is bound to the cytosolic domain of the enzyme. Figure 3 and Figure S4 illustrate this interaction in terms of the protein-ligand hydrogen (h-) bonds, which are stable during all MD simulations, although some key interactions vary compared to the reference crystal structure [19]. In particular, Arg^246^, which does not bind CoA in the experimental structure, strongly interacts with pyrophosphate and adenine moieties in all MD trajectories (Figure 3A,B). (It is worth noting that in the crystal structure Arg^246^ along with Arg^126^ coordinate via salt bridges a buffer phosphate ion, which was excluded from our model systems.) Interestingly, Lee et al. [19] were not satisfied with the experimental structure and additionally refined it using MD, revealing many peculiarities that we also observe in our trajectories. For example, in MD Arg^246^ reveals a π-cation interaction with CoA’s adenine moiety [19], which was also detected here across the most of the calculated trajectories (except for C16^(2)^; see Table 1), reaching the maximum lifetime of 0.95 (95% of MD states in MD trajectories with C18 and C20). The distal phosphate group forms a h-bonds network with Lys^135^, His^140^, His^141^, and Ser^143^ for most of the time. The pyrophosphate moiety can be coordinated by Arg^246^ and Ser^143^ (Figure 3, Figures S4 and S5).

The observed inconsistencies in the ways of CoA head’s binding between our MD results and the reference experimental structure of the mutant hDHHS20 dimer [19] may result from the dimeric structure of crystallized protein, while the native functional state is monomeric. The authors of work [19] claim that the aberrant dimerization may have led to a distorted complex, and performed an MD refinement, which revealed the R state (reactive in [19]). Eventually, most of the peculiarities from our MD simulations are consistent with the computational results of Lee et al.; but not all of them—e.g., we do not observe the 50/50 balance between R and NR (nonreactive in [19]) conformations (rather, we have mostly reactive ones); also, we do not observe the acyl-CoA’s carbonyl group fixation by His^231^, whereas in our simulations this group is mostly bound by conserved Trp^158^.

## 3. Discussion

The family of DHHC-acyltransferases has attracted more and more attention in recent years in the context of viral infection [13,14,15,16,22]. Several representatives of the family including hDHHC20 were proposed to modify influenza A virus hemagglutinin and M2 protein [13], as well as Spike protein of SARS-CoV-2 [14,15]. Despite there is no doubt that binding of fatty acid tails to conserved cysteines is crucial for membrane fusion, entry and/or assembly of many enveloped viruses [23,24], structural mechanisms and functional consequences of this lipid modification are poorly understood.

In particular, it is absolutely unknown what is the structural basis for the so-called differential S-acylation with various types of fatty acids [10], e.g., palmitates (C16:0) or stearates (C18:0) found by us using MALDI-TOF mass spectrometry for spike proteins from various families of enveloped viruses [7,25,26,27]. Binding C18- vs. C16-tail may actually have serious structural impact, since the difference in their hydrophobicity is essential, and fine-tune structural stability of homotrimeric spike complexes as we proposed earlier for influenza A virus hemagglutinins [7].

One of the mechanisms of differential S-acylation may be the lipid selectivity of the DHHC-enzymes. Earlier, a biochemical study revealed several DHHCs involved in S-acylation of influenza A virus hemagglutinin: ZDHHC2, 8, 15 and 20 [13]. The question remains: whether all of them can transfer any type of fatty acids to any acylation site or, alternatively, the certain enzymes preferentially transfer specific types of fatty acids onto particular acylation sites.

In this paper, we have left aside the structural features of the protein substrate and the issue of concentration in the membrane/cell compartments and the availability of the necessary metabolites for the acylation reaction. Instead, we continued a detailed study of the topological compatibility of the internal cavity of the enzyme and its lipid substrate using the example of hDHHC20 and a series of acyl-CoAs with various aliphatic tails. We found for the first time that hDHHC20 exhibits preference for C16-CoA over other types of the full-size lipid substrates (C12, C14, C18, C20)-CoAs proving the geometrical fitness of its inner cavity. Moreover, this substrate exhibited the maximal reactivity in relation to hDHHC20 as assessed by a simple geometric criterion of nucleophilic attack we have developed. Similar preference of C16-MEA, a shortened analog of full-size C16-CoA, for hDHHC20 was found by us earlier [18]. This preference shifted to C14-MEA in case of S29F, and to C18-MEA in case of Y181A mutants of hDHHC20 [18] that is in accordance with biochemical data [11]. In aggregate, our MD simulations demonstrate that the “ceiling” of the inner cavity of the enzyme is a limiting geometrical barrier affecting the enzyme’s physicochemical properties. Thus, clear preference of hDHHC20 for C16- indicates that the enzyme’s lipid specificity may participate in providing differential fatty acylation of proteins.

It can be assumed that other DHHC-enzymes better accept longer or shorter fatty acid residues. In the future, it would be interesting to study the fitness and reactivity of a panel of full-size lipid substrates with other DHHCs using the developed protocols. However, this should be preceded by validation of the modelled spatial structures of these enzymes due to the absence of direct experimental data.

## 4. Materials and Methods

The starting models of the full-length hDHHC20 complex with various acyl-CoA molecules were derived from the available crystal structure (PDB entry 7KHM) [19], in which catalytically inactive Ser^156^ was reversed back to Cys^156^ using the standard *mutagenesis* option of the Pymol software v. 2.5.0 [28]. Five MD systems were generated that differed in the length of the substrate’s acyl chain: C12, C14, C16, C18, and C20, all in triplicate (Table 1). The representative trajectory from every group was designated with ^(1)^ and used for calculation of the most of the results, if other is not stated. Resulting models of the complexes were immersed into a pre-equilibrated lipid bilayer (288 1-palmitoyl-2-oleoyl-sn-phosphatidylcholine (POPC) molecules in order to mimic the plasma membrane [29], where DHHC20 is mainly localized [30]); overlapping phospholipids were removed. The bilayer-embedded complexes were placed into rectangular boxes (typical size of 90 × 90 × 135 Å^3^) and solvated with explicit water (TIP3P model [31]). Na^+^ and Cl^−^ ions [32,33] were added to the solvent box to correspond to 150 mM NaCl.

The simulated systems were first equilibrated in several stages: 5000 steps of steepest descent minimization followed by heating from 5 K to 310 K during a 5-ns MD run, in which internal coordinates of the protein and ligand heavy atoms were restrained to permit membrane relaxation. Then, the systems were subjected to long-term 500 ns MD simulations without restraints.

MD simulations were carried out with the GROMACS software package version 2020.4 [34] using the CHARMM36 force field [35,36,37,38,39]. An integration time step of 2 fs was used and 3D periodic boundary conditions were imposed. Simulations were performed with a constant temperature (310 K) and pressure (1 bar) maintained using the V-rescale [40] and the Parrinello–Rahman algorithms [41], respectively. The semi-isotropic pressure coupling in the bilayer plane and along the membrane normal was used in the simulations. The 12 Å cutoff radius was defined for the Coulombic and van der Waals interactions. Electrostatic interactions were evaluated using the particle-mesh Ewald (PME) summation [42] (real space cutoff of 12 Å). Protein along with membrane lipids and solvent molecules were coupled separately.

Atomic coordinates from MD trajectories were centered on the protein molecule and analyzed with a timestep of 10–100 ps using original GROMACS and in-house utilities. The density profiles, atomic distances and coordinates were extracted using *gmx density*, *gmx dist* and *gmx traj* GROMACS utilities, respectively. Intermolecular contacts, including hydrogen bonds, were calculated using GROMACS (*gmx hbond*) and in-house software. The distance cut-off for intermolecular acyl chain/protein contacts (Figure S1) was 4 Å. Molecular graphics were rendered using PyMOL v. 2.5.0 [28] and UCSF Chimera v. 1.11.2 [43].

## 5. Conclusions

In this work, structural and dynamic modeling of the hDHHC20 complex with a full-size acyl-CoA, bearing acyl chains of various lengths, was carried out for the first time. It was shown that the acyl chain is located in the hydrophobic cavity of the enzyme in such a way that its terminal methyl group rests against the cavity ceiling, regardless of its length. Acyl chains inside the hydrophobic cavity did not distort and remained elongated, and the acyl-CoA heads did not enter the hydrophobic region of the membrane, in contrast to the shortened substrate (acyl-MEA) [18]. Using a simple geometric criterion of reactivity of the DHHC/acyl-CoA complexes conformations, we demonstrated that C16-CoA is the most capable of acylating hDHHC20. This explains the selectivity profile of the enzyme.

It was earlier proposed that DHHCs required for acylation of influenza hemagglutinin could be promising drug targets, since their blockade should result in suppression of viral replication, while acylation of cellular proteins will be barely affected [44,45]. Blocking DHHC20, which acylates S-protein, is proposed to combat SARS-CoV-2 [14,22]. Consequently, the MD simulations of DHHC-enzymes in complex with full-size CoA substrates described here seems to be a valuable tool for future design of fatty acid-based inhibitors to prevent autoacylation of different DHHCs. This class of lipids is yet to be employed as antiviral inhibitors—the only examples to date are linoleic acid, which stabilizes a locked S-protein conformation [46] to inhibit SARS-CoV-2 replication; and a designed palmitic acid-based lipopeptide fusion inhibitor [47].

A perspective goal of this work is to extend the developed MD-based protocols to study other DHHCs and, specifically, search for enzymes specific to longer (un)saturated fatty acids (e.g., C18:0 or C18:1), which may regulate mitochondria in vivo in humans [48] and are involved in oncogenic signaling downstream of growth factors [5]. In addition, some viral proteins such as hemagglutinin-esterase-fusion of influenza C virus, F-protein of Newcastle disease virus as well as E1 of Semliki Forest virus bear primarily stearates, not palmitates [26], and identification of the responsible enzymes will help to advance our search for potential antiviral inhibitors.

## Data Availability

The data presented in this study are openly available in the Zenodo archive (10.5281/zenodo.7334476, accessed on 23 November 2022).

## Supplementary Materials

The following supporting information can be downloaded at: https://www.mdpi.com/article/10.3390/ijms232314791/s1.
