# Supplementary material for: The Mechanism of Selective Recognition of Lipid Substrate by hDHHC20 Enzyme
Source: Int J Mol Sci. 2022 Nov 26;23(23):14791. doi: 10.3390/ijms232314791 (PMC9739150; doi:10.3390/ijms232314791)
Supplement: Supplementary file 1 [file ijms-23-14791-s001.zip › ijms-2012422-supplementary.pdf]

Supplementary information

The mechanism of selective recognition of lipid substrate by hDHHC20 enzyme

Irina S. Panina[1,2], Nikolay A. Kyrlov[1,2], Anton O. Chugunov[1,2,3,]* Roman G. Efremov[1,2,3], Larisa V. Kordyukova[4]

[1]Shemyakin-Ovchinnikov Institute of Bioorganic Chemistry, Russian Academy of Sciences, Moscow, Russia

[2]National Research University Higher School of Economics, International Laboratory for Supercomputer Atomistic Modelling and Multi-Scale Analysis, Moscow, Russia

[3]Moscow Institute of Physics and Technology (State University), Dolgoprudny, Moscow Region, Russia

[4]Belozersky Institute of Physico-Chemical Biology, Lomonosov Moscow State University, Moscow, Russia

*Corresponding author. Email: batch2k@yandex.ru

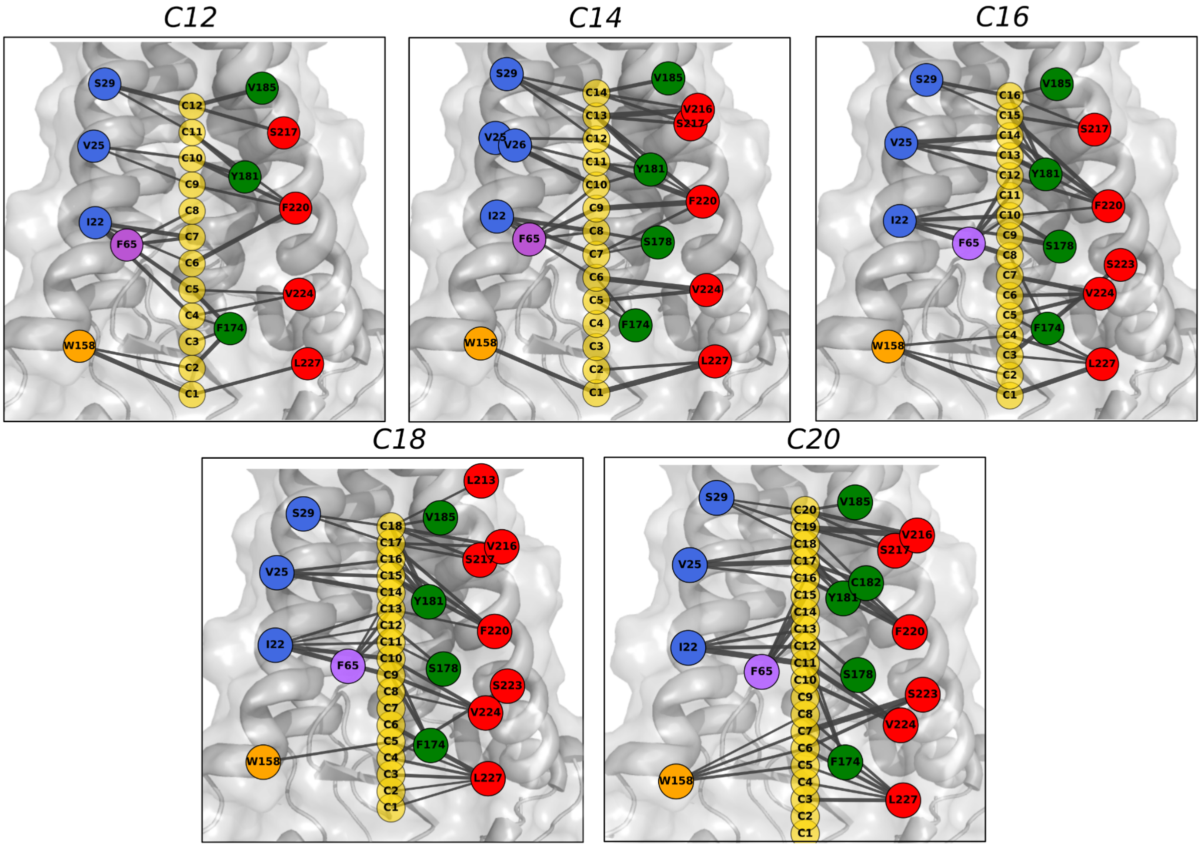

Supplementary Figure S1. Acyl-CoA fatty residue contacts in hDHHC20 cavity during MD. Amino acid residues that form contacts with atoms C1–C16 of the ligand (*yellow*) for >50% of MD time are indicated and color-coded according to location in different TMHs: TMH-1 — *blue*; TMH-2 — *purple*; TMH-3 — *green*; TMH-4 — *red*. W158 near the active site is *orange*. It presumably forms the "entrance gate" to the enzyme cavity and often interacts with the lower part of the acyl tail. Linewidth of a contact is proportional to its lifetime. Interestingly, residues of TMH-2 interact least with the substrate, despite the abundance of large aromatic residues facing the cavity.

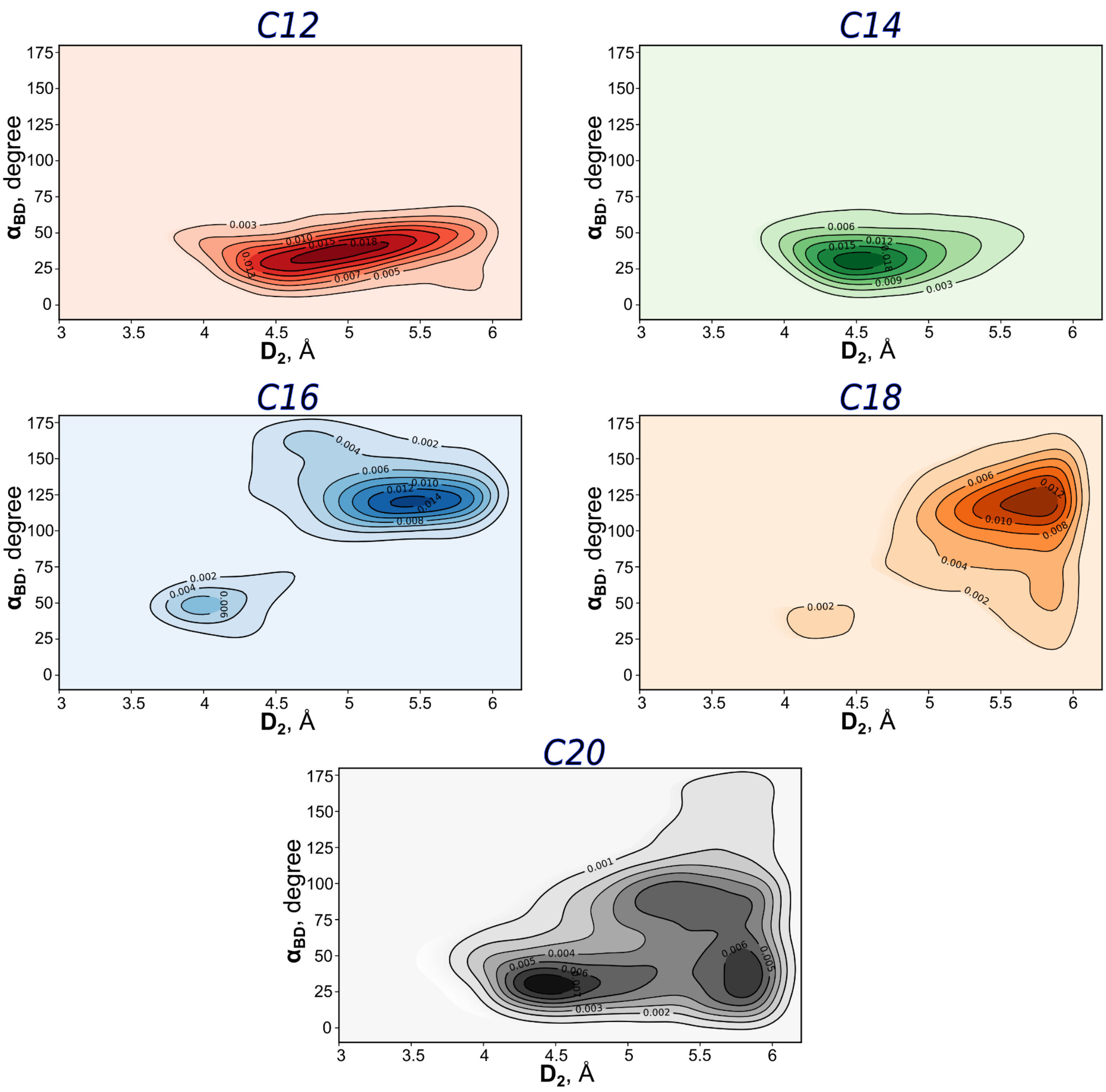

Supplementary Figure S2. Reactivity of the enzyme–substrate complex during MD. Each panel (C12–C20) is contoured 2D distribution of probability of the states, described by (1) the distance between S atom of the catalytic Cys[156] and carbonyl C atom of acyl residue (D_2) and (2) the Bürgi–Dunitz angle ($α_{BD}$) of nucleophilic attack (see Fig. 2 for description of the parameters). Only $D_2 < 6$ Å parts of all trajectories (replicas from the Table 1 joined) are shown here. We treat as reactive states with $α_{BD} > 90°$: note that C16 is the most reactive, then comes C18, where notable part of the states has $α_{BD} < 90°$.

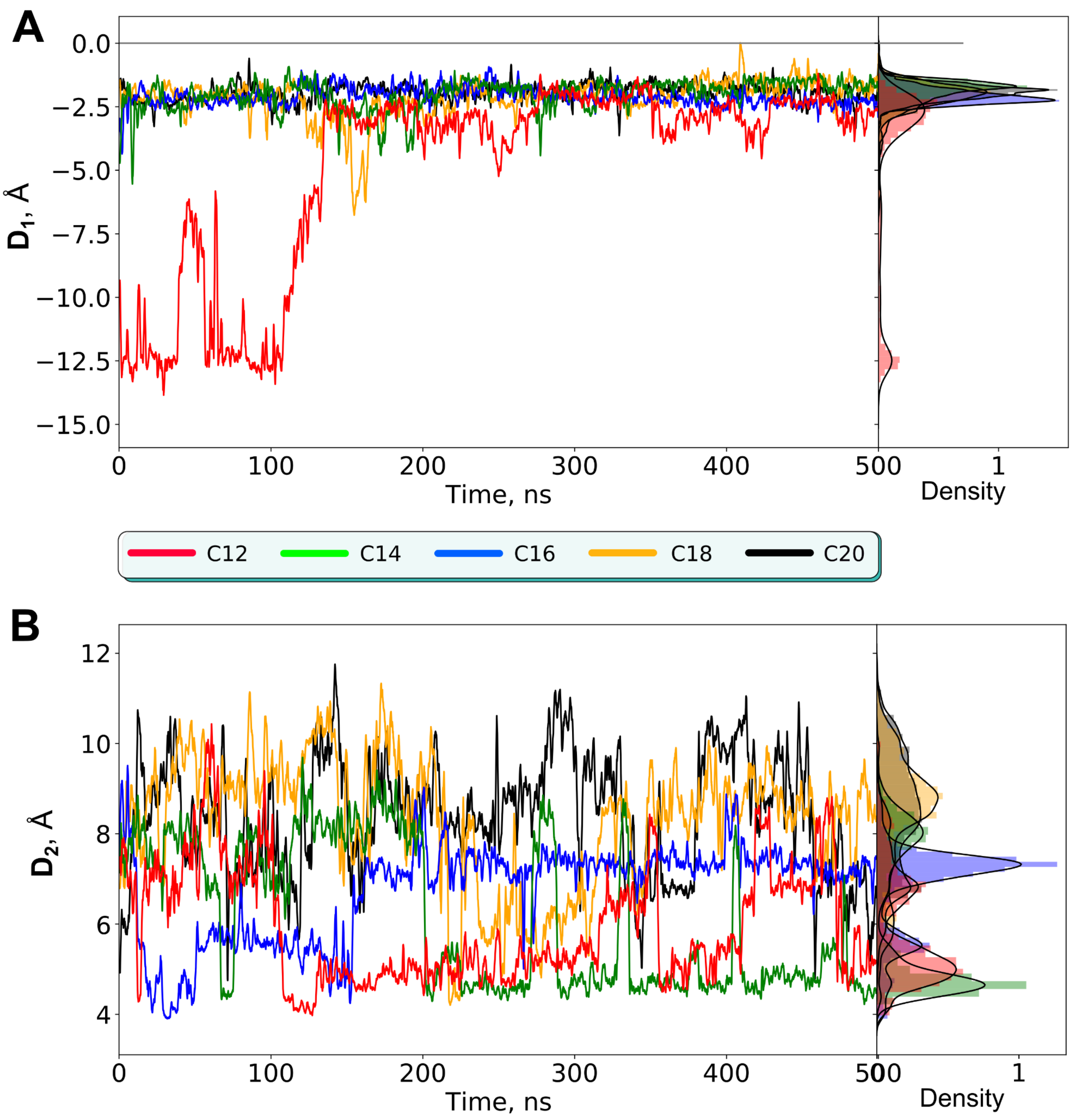

Supplementary Figure S3. Time-averaged (in a 0.1 ns window) dynamic change of D_1 (A) and D_2 (B). To the right, probability density functions are provided for a corresponding time series. Calculations are performed on the representative set of trajectories and colored according to the *legend*. **A.** $D_1 = 0$ is the ceiling level. Rare spike-like drops of D_1 correspond to the partial exit of the substrate from the cavity. **B.** D_2 under 6 Å (characteristic for a van-der-Waals contact) is one of the criteria of reactive states (see Fig. S2). Note that, although C12 and C14 may have low D_2 values, these states cannot be considered as reactive due to the sharp angle of the nucleophilic attack (α_{BD}; see Fig. S2).

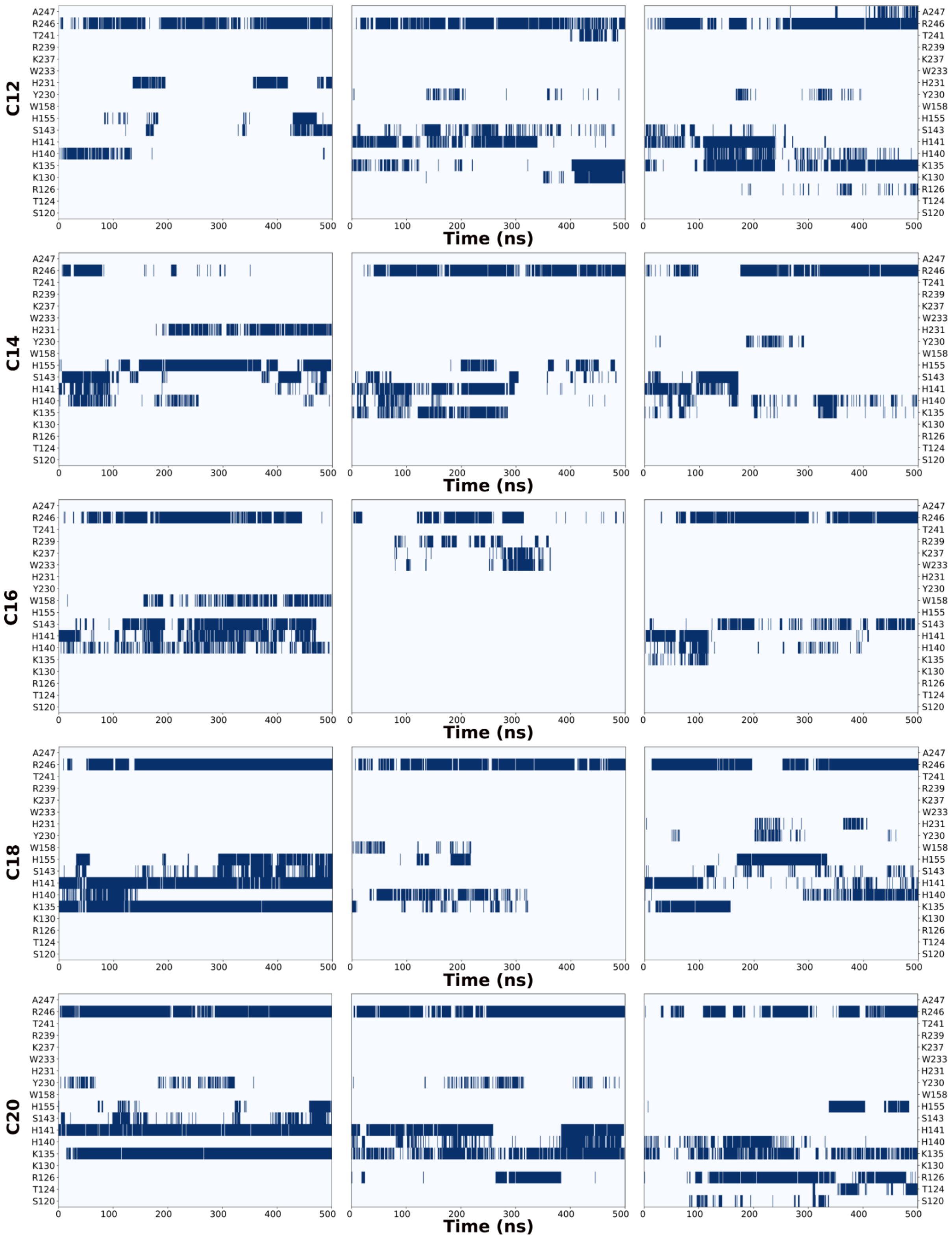

Supplementary Figure S4. H-bonding in the hDHHC20/acyl-CoA complexes. Each panel is a h-bond map between hDHHC20 and polar CoA head for given acyl chain length (C12–C20), in three replicas ((1)–(3), according to the Table 1). Blue hatches indicate h-bond between CoA and particular hDHHC20 residue (along the vertical axis) at a given MD time (along the horizontal axis). Only residues that form h-bonds with >10%-lifetime in at least one trajectory, are shown.

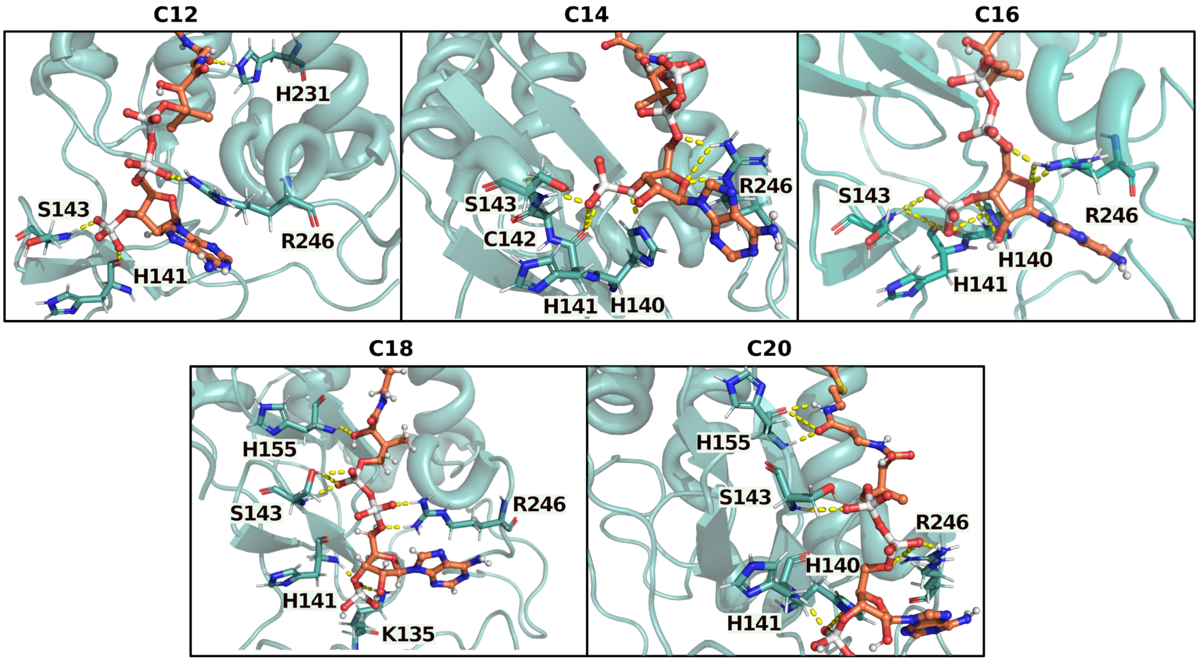

Supplementary Figure S5. Final snapshots of the complexes after the MD, zoomed at the CoA head binding site. The binding partners are shown as following: hDHHC20 — *green ribbons*; C16-CoA— *orange balls & sticks*. Amino acid residues that bind acyl-CoA by h-bonds (*yellow dashes*) in MD structure, are shown with sticks.
